# Enhancing the effectiveness of multidisciplinary interventions to improve blood culture efficiency and optimize antimicrobial utilization

**DOI:** 10.3389/fpubh.2026.1903984

**Published:** 2026-07-24

**Authors:** Rana Hassan D. Almaghrabi, Deena S. S. Alharbi, Khulud Salem Mohammed Alqahtani, Muneerah Abdulrahman Y. Alzouman, Turki Saqer J. Almutairi, Abdullah Bandar T. Alotaibi, Mohamed Gafir Rajab, Maher Abdrabalredah Mohammed Al Kuwaiti, Amal Ibrahim Zamil Akresh, Noura S. Alaboudi, Nour M. AlThibani, Adel Saeed S. Alotaibi, Naoufel M. Kaabia, Maaly Zayed Eid Alotaibi, Saad Saeed Tamam Alalyani, Wafa Ahmed Mohammed Alfahad, Fatimah Alzahra Abdulkarim A. Alnakhli, Asma Abdullah I. Alabdulsalam

**Affiliations:** 1 Antimicrobial Stewardship Center of Excellence, Prince Sultan Military Medical City (PSMMC), Riyadh, Saudi Arabia; 2 Central Military Laboratory and Blood Blank, PSMMC, Riyadh, Saudi Arabia; 3 Executive Nursing affairs, PSMMC, Riyadh, Saudi Arabia; 4 Department of Emergency, PSMMC, Riyadh, Saudi Arabia; 5 Department of Pharmceutical Services, PSMMC, Riyadh, Saudi Arabia; 6 Infection Prevention and Control Center of Excellence, PSMMC, Riyadh, Saudi Arabia; 7 Department of Continuous Quality Improvement and Patient Safety, PSMMC, Riyadh, Saudi Arabia

**Keywords:** antimicrobial stewardship, blood culture, contamination, diagnostic stewardship, emergency department

## Abstract

**Background:**

Blood cultures are essential for diagnosing bloodstream infections and for guiding antimicrobial therapy. Excessive blood culture utilization in non-ICU settings can lead to false-positive results, antibiotic exposure, increased healthcare costs, and harm to patients. Diagnostic stewardship (DS) aims to improve the appropriateness of diagnostic testing and optimize antimicrobial use; however, its implementation remains inconsistent across healthcare systems.

**Methods:**

This prospective quasi-experimental study evaluated a multidisciplinary stewardship program in adult and pediatric emergency departments. From May to August 2025, with baseline data from January to April 2025 used for comparison. Improving specimen collection practices to reduce contamination, implementing clinical guidance to optimize blood culture ordering, and strengthening antimicrobial stewardship by monitoring Days of Therapy (DOT). Data were extracted from electronic health records and analyzed using descriptive statistics. Blood Culture Event Rate (BCER), contamination rates, antimicrobial utilization, and 30-day emergency department readmissions.

**Results:**

The Blood Culture Event Rate (BCER), decreased from 12 to 4% in adults and from 4 to 1% in pediatric patients. Blood culture contamination declined by approximately 87%, decreasing from approximately 35 to fewer than five cases per month. Antimicrobial utilization improved, with a 100% reduction in ceftazidime/avibactam use and 40.39% reduction in Meropenem DOT, which is the aggregate number of days on which a patient received at least one dose of a specific antimicrobial agent. Additional reductions were observed for ceftazidime (61%), ciprofloxacin (29.49%), and vancomycin (19.83%). Compliance with blood culture ordering guidelines has improved from 70 to 97%, and no blood culture-related 30-day readmissions have been reported.

**Conclusion:**

A multidisciplinary diagnostic stewardship approach significantly improved blood culture utilization, reduced contamination, and optimized antimicrobial prescriptions.

## Introduction

Blood cultures are a cornerstone in diagnosing sepsis and bloodstream infections, facilitating targeted antimicrobial therapy, and guiding further clinical management. However, excessive use, particularly in low-risk cases, can lead to false-positive results, unwarranted antibiotic treatment, elevated healthcare expenditure, and potential patient harm (1). These issues are especially evident in non-ICU inpatient settings, where true bacteremia is uncommon and contamination rates often exceed acceptable thresholds (2). In such scenarios, contaminated results can prompt unnecessary procedures, prolonged hospitalization, and additional interventions. The blood culture event rate (BCER) per 100 admissions or patient-days is a core metric in diagnostic stewardship, reflecting the appropriateness of utilization. A high BCER may indicate over-testing, whereas a reduced BCER suggests improved diagnostic precision and resource optimization, particularly in high-throughput settings such as emergency departments (1, 3).

Diagnostic stewardship (DS) can effectively curb unnecessary blood culture use, although formal guidelines for specific clinical scenarios are lacking. Engaging multidisciplinary teams, including infectious disease specialists, can optimize cultural utilization and monitor appropriate use (4). Structured initiatives have demonstrated that targeted interventions can safely reduce unnecessary collection. For instance, a clinical algorithm for fever or instability safely reduces blood culture use without increasing sepsis, mortality, or readmissions while also curbing broad-spectrum antibiotic use (2). At Johns Hopkins Hospital, a DS program reduced blood culture rates by 18% in the ICU and 30% in medical units without increasing mortality or readmissions (5). The Pediatric Bright STAR Collaborative developed 19 consensus-based recommendations to reduce unnecessary blood cultures in critically ill children (6, 7). Subsequent implementation across 14 pediatric intensive care units demonstrated significant reductions in both blood culture rates and broad-spectrum antibiotic use without any increase in sepsis, mortality, or hospital readmissions (6, 7). This underscores the value of structured multidisciplinary stewardship. However, the adoption of more sensitive blood culture systems, while improving pathogen detection, has introduced a trade-off: rising contamination rates that surpass the national threshold of ≤3% in various high-acuity settings (8). This increase in false positives highlights the need for a DS that balances enhanced sensitivity with clinical utilization and resource efficiency (9).

Despite growing evidence, inappropriate blood culture practices persist in non-ICU settings, where low bacteremia rates and high contamination challenge clinical value. Although interventions such as algorithms and multidisciplinary programs have shown promise, their impact varies across institutions. At our tertiary care hospital, a strategic multidisciplinary initiative was launched to refine blood culture protocols and strengthen antimicrobial stewardship. This prospective study evaluated targeted interventions across high-throughput emergency departments, integrating expertise from Emergency Medicine, Microbiology, Pharmacy, Nursing, and Infection Control. The goal is to improve diagnostic precision, reduce contamination, and provide scalable, context-specific clinical workflows.

To our knowledge, few studies have evaluated multidisciplinary diagnostic stewardship interventions targeting both blood culture utilization and antimicrobial consumption simultaneously in adult and pediatric emergency departments within a Middle Eastern tertiary-care setting. This study contributes real-world evidence regarding the implementation and impact of a multidisciplinary emergency department-focused stewardship strategy.

Emergency departments represent a unique setting for diagnostic stewardship because clinical decisions must be made rapidly, often before definitive diagnostic information becomes available. Consequently, blood cultures may be ordered defensively or routinely, increasing the risk of overutilization, contamination, and unnecessary antimicrobial exposure. Therefore, interventions targeting emergency departments may provide substantial opportunities to improve diagnostic efficiency and antimicrobial stewardship.

This study aimed to evaluate whether a multidisciplinary diagnostic stewardship intervention implemented in adult and pediatric emergency departments was associated with reductions in blood culture utilization, blood culture contamination, broad-spectrum antimicrobial use, and blood culture-related 30-day readmissions.

### Knowledge gap

Although previous studies have demonstrated the benefits of diagnostic stewardship in inpatient and critical care settings, evidence regarding multidisciplinary blood culture stewardship programs in adult and pediatric emergency departments remains limited, particularly in the Middle East. Furthermore, the impact of such interventions on both blood culture utilization and antimicrobial consumption has not been adequately evaluated in this context.

## Method

This prospective quasi-experimental before-and-after quality improvement study was conducted in the adult and pediatric emergency departments of Prince Sultan Military Medical City (PSMMC), Riyadh, Saudi Arabia. Outcomes during the intervention period (May–August 2025) were compared with those during the baseline pre-intervention period (January–April 2025).

The initiative engaged key stakeholders in Antimicrobial Stewardship, Emergency Medicine, Clinical Microbiology, Infection Prevention and Control, Pharmacy Services, and Nursing, each contributing to enhanced diagnostic accuracy, antimicrobial stewardship, and patient safety within an integrated care framework. Ethical approval was obtained from the Research Ethics Committee of PSMMC in Riyadh, KSA, before study initiation. Initiative selection was guided by strategic alignment with the tertiary care hospital’s long-term mission, prioritizing projects that support institutional growth, clinical excellence, and responsiveness to evolving patient and staff needs. Efforts that lacked stakeholder engagement, operational feasibility, or strategic relevance were excluded to ensure clinical soundness and sustainability. Appropriateness was defined according to the institutional blood culture indication algorithm. Two infectious diseases physicians independently reviewed randomly selected blood culture requests to assess compliance with the guideline.

This study synthesized global best practices with localized implementation, addressing a critical gap in DS tailored to non-ICU settings. Data collection focused on the following key variables: blood culture rates, contamination rates, specimen collection timing, antimicrobial prescription patterns, and Days of Therapy (DOT). Blood culture contamination was defined as isolation of common skin flora from a single blood culture set in the absence of clinical evidence of bloodstream infection, consistent with institutional microbiology criteria.

Only non-ICU cases were included to reflect emergency department workflows. Interventions targeted three domains: improving specimen collection protocols to reduce contamination, optimizing antimicrobial prescriptions with emphasis on DOT, and enhancing workflow coordination to minimize unnecessary blood cultures. These changes were introduced in phases, enabling a comparison between pre- and post-intervention periods.

### Primary outcomes

Blood Culture Event Rate (BCER) per 100 emergency department admissions.

Blood culture contamination rate.

### Secondary outcomes

Antimicrobial utilization is measured using Days of Therapy (DOT).

Appropriateness of blood culture requests according to institutional blood culture guidelines.

Blood culture-related 30-day emergency department readmissions.

All adult and pediatric patients attending the emergency department during the study period for whom blood cultures were ordered are included in this study. Patients admitted directly to intensive care units, Duplicate blood cultures obtained within the same clinical episode, and Blood cultures collected outside the emergency department are excluded from this study.

Diagnostic Stewardship Intervention in Three Phases:

Phase 1 (May 2025):

Blood culture indication algorithm introduced, and Educational sessions for physicians.

Phase 2 (June 2025):

Staff education for nurses/phlebotomists and the standardized collection technique.

Phase 3 (July–August 2025): Prospective audit and feedback, Blood culture appropriateness review, and Antimicrobial utilization monitoring.

### Statistical analysis

Statistical analyses were conducted using SPSS Statistics (version 22.0; IBM). Descriptive statistics were used to summarize categorical variables as frequencies and percentages, and continuous variables as means with standard deviations. Pre- and post-intervention comparisons were performed to assess the changes in blood culture rates, contamination rates. Missing data were reviewed and excluded from analyses when incomplete documentation prevented outcome assessment.

### Ethical consideration

Ethical approval for this study was obtained from the Scientific Research Center of Prince Sultan Military Medical City (PSMMC), Riyadh, Saudi Arabia. (Approval No: E-2641). The datasets generated and analyzed during the current study are not publicly available because of institutional and patient confidentiality policies.

## Results

**Figure 1:**
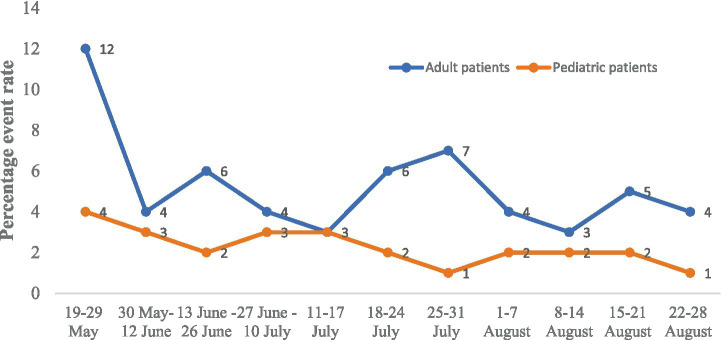
Trend in blood culture event rate (BCER) per 100 admissions before and after a diagnostic stewardship intervention in adult and pediatric emergency departments.

The diagnostic stewardship initiative at a tertiary care hospital, implemented across pediatric and adult emergency departments, has yielded measurable improvements in blood culture practices and antimicrobial utilization. Key achievements included the uninterrupted availability of blood culture bottles, reduced antimicrobial use, decreased contamination rates, and the absence of adverse effects related to blood culture interventions. Between May and August 2025, both adult patients and pediatric emergency units demonstrated a consistent and encouraging decline in Blood Culture Event Rate (BCER) per 100 emergency admissions. The adult patient unit accounted for 12%, whereas the pediatric unit accounted for 4%. Over the following weeks, both units showed steady reductions, ultimately reaching 1% in the pediatric unit and 4% in the adult unit by late August. A comparative analysis of the broad-spectrum antibiotic consumption between May and August revealed notable reductions across all agents, as shown in Figure 1.

**Figure 2:**
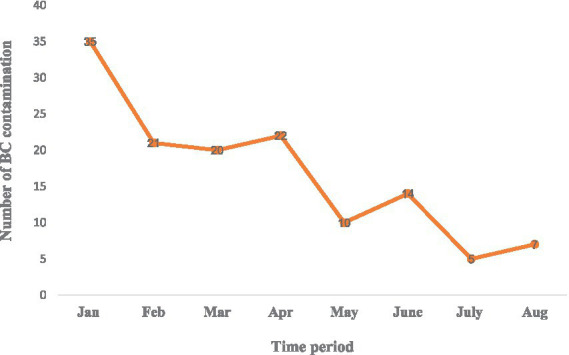
Reduction in monthly blood culture contamination events following a diagnostic stewardship intervention.

Blood culture contamination rates declined markedly following the stewardship intervention, starting from a peak in January 2025, dropped sharply, and stabilized at a significantly lower baseline between May and August. Contaminated cultures decreased from approximately 35 in May to 5 in July. Notably, up to 50% of positive cultures had previously been contaminated, underscoring the impact of improved collection techniques and targeted staff education, as shown in Figure 2.

The most substantial percentage decrease was observed for Ceftazidime/Avibactam, which dropped from 2.8 to 0, reflecting a 100% reduction. Ceftazidime use declined by 60.98% (from 12.3 to 4.8), followed by meropenem (40.39%) decrease (35.9 to 21.4). Ciprofloxacin and Vancomycin showed reductions of 29.49 and 19.83%, respectively. Piperacillin/tazobactam decreased by 14.12% (96.3 to 82.7), whereas amoxicillin/clavulanate exhibited the smallest change at 7.36% (25.8 to 23.9) (Table 1).

**Table 1:** Change in days of therapy (DOT) for key broad-spectrum antibiotics following a diagnostic stewardship intervention.

Broad-spectrum antibiotics	DOT in May	DOT in August	Percentage change
Ceftazidime/avibactam	2.8	0.0	−100.0%
Ceftazidime	12.3	4.8	−61.0%
Meropenem	35.9	21.4	−40.4%
Ciprofloxacin	7.8	5.5	−29.5%
Vancomycin	47.4	38.0	−19.8%
Piperacillin/tazobactam	96.3	82.7	−14.1%
Amoxicillin/clavulanate	25.8	23.9	−7.4%

The appropriateness of blood culture requests was assessed in two phases, following the implementation of clinical guidelines, an indication algorithm, and awareness initiatives targeting nurses, phlebotomists, and other relevant staff. In the first phase, appropriateness was measured based on documentation. Initially, the documentation-based appropriateness rate was 70%, which increased to 97% in the second phase, indicating a substantial improvement in guideline compliance. Second, an expert team was formed to validate the appropriateness of blood culture requests beyond documentation alone by reviewing whether the cultures were clinically justified.

Thirty-day emergency department readmissions were monitored as safety indicators. While readmissions occurred, none was attributed to blood culture-related decisions, confirming the safety of the stewardship approach. These findings are consistent with published evidence showing that DS can reduce blood culture utilization by 30–40%, lower contamination rates, improve positivity rates, and decrease broad-spectrum antibiotic use without increasing mortality, length of stay, or readmissions.

Table 2 results indicated the primary and secondary outcomes before and after implementation of the diagnostic stewardship intervention. Blood culture utilization decreased substantially in both adult and pediatric emergency departments. The Adult Blood Culture Event Rate (BCER) decreased from 12 to 4 per 100 emergency department admissions, representing a 66.7% reduction. Similarly, the Pediatric BCER decreased from 4 to 1 per 100 emergency department admissions, corresponding to a 75.0% reduction. These findings suggest a marked decrease in blood culture ordering following implementation of stewardship measures.

**Table 2:** Primary and Secondary outcomes before and after diagnostic stewardship intervention.

Outcome	Baseline	Intervention	Relative change
Adult BCER (per 100 ED admission)	12	4	−66.7
Pediatric BCER (per 100 ED admission)	4	1	−75.0
Blood culture contamination events (monthly)	35	5	−85.7
Documentation-based appropriateness	70	97	+38. 6

Blood culture contamination events also declined considerably, decreasing from 35 to 5 events per month, representing an 85.7% reduction. This improvement is consistent with enhanced specimen collection practices, staff education, and adherence to standardized blood culture collection protocols. Documentation-based appropriateness of blood culture requests improved from 70% at baseline to 97% during the intervention period, representing a 38.6% relative increase. This finding indicates improved compliance with institutional blood culture ordering guidelines and documentation standards following implementation of the multidisciplinary stewardship intervention.

To Calculate the Blood culture contamination used contaminated culture numbers and divided by total blood culture and multiplied by 100, the Blood culture contamination decreased substantially following implementation of the diagnostic stewardship intervention. During the baseline period, 35 contaminated cultures were identified among 820 blood cultures collected, corresponding to a contamination rate of 3.9%. Following intervention implementation, contamination events decreased to 5 among 510 blood cultures, corresponding to a contamination rate of 1.0%. This represented a relative reduction of 76.7% in contamination rate. The reduction was temporally associated with implementation of standardized specimen collection procedures, targeted staff education, and enhanced multidisciplinary diagnostic stewardship practices.

## Discussion

The Diagnostic Stewardship Initiative in the emergency department of a tertiary-care hospital in Riyadh, KSA, demonstrated that targeted multidisciplinary interventions can significantly improve blood culture efficiency and optimize antimicrobial utilization in a high-throughput, non-ICU setting. Our findings underscore the necessity of integrating DS as a fundamental component of effective AMS, aligning the diagnostic pathway (“right test, right patient, right action”) with subsequent therapeutic management.

The introduction of DS protocols resulted in a pronounced and clinically significant reduction in the BCER per 100 across both ED units. The adult patient unit saw its BCER decline from 12 to 4%, whereas the pediatric unit decreased from 4 to 1% per 100 emergency admissions (Figure 1). This performance is highly relevant when compared to established global benchmarks. The baseline BCER of 12% in our tertiary care hospital’s adult patient emergency unit closely mirrored the pre-intervention rate of 12.17 per 100 ED admissions reported in the Duke University ED study. However, our hospital achieved a greater proportional reduction in the overall blood culture utilization. The effectiveness of this algorithm-based approach supports findings from other high-acuity settings: adult inpatient DS programs have documented blood culture rate reductions of 18% in the medical intensive care unit and 30% in medicine units^2^, while the pediatric Bright STAR collaborative demonstrated that utilization can be safely reduced by 30–40% in critically ill children (7). In another study, the implementation of a blood culture algorithm led to a decrease in BCER from 12.17 to 10.50 per 100 ED admissions, further validating the impact of targeted stewardship interventions (1). Blood culture stewardship has been implemented in more than 20 hospitals in the U.S. and U.K. as part of BrighT STAR, a JHCC-led DS collaborative that has safely reduced the number of blood cultures collected with no increase in sepsis, septic shock, mortality, length of stay, or readmissions. Additionally, these clinical decision support tools reduced the use of broad-spectrum antibiotics by 13% (7).

The substantial reduction in blood culture contamination, decreasing from approximately 35 events in January 2025 to around 5 by July 2025, validates the efficacy of targeted DS focusing on the preanalytical phase. This improvement reflects the impact of implementing improved collection protocols and dedicated staff education (Figure 2). Addressing contamination is paramount because up to 50% of the previously positive cultures were identified as contaminants. Achieving this lower baseline minimizes the risk of false-positive results, thereby enhancing diagnostic precision, reducing unwarranted antibiotic exposure, and optimizing antimicrobial utilization without compromising patient safety. Our findings corroborate previous quality improvement projects reporting that 60% of blood culture contaminations occur during the pre-analytical phase and that targeted interventions reduce contamination rates by 30% over a defined period (10).

The assessment of blood culture appropriateness following the implementation of clinical guidelines, an indication algorithm, and targeted awareness campaigns demonstrated the effective application of diagnostic stewardship. Documentation-based appropriateness improved from 70 to 97%, indicating rapid staff compliance during the pre-analytical phase. This initial success laid the groundwork for a subsequent expert review that assessed clinical justification beyond documentation, thereby reinforcing the core stewardship principle of ordering the right test for the right patient at the appropriate time. Globally, inappropriate blood culture remains a persistent challenge, particularly in high-throughput EDs, where up to 90% of cultures yield no organism growth. Comparative data show that only 62% of BCx requests were appropriate in a community hospital ED and only 50% in academic medicine units, underscoring the widespread need for targeted interventions (11). This outcome highlights the effectiveness of pre-analytical phase interventions, including improved specimen collection protocols, communication, and education provided to trained phlebotomists and nurses. Achieving a significant reduction in contamination rates is recognized as a key goal of DS, as evidenced by other studies demonstrating successful reductions in contamination.

A multidisciplinary intervention conducted in a Chinese tertiary hospital was reported to enhance blood culture efficiency, including reducing contamination from 1.4 to 0.9% (9). The dramatic reduction in broad-spectrum antimicrobial DOT, highlighted by the 100% elimination of ceftazidime/avibactam and the 40.39% decrease in meropenem, reflects the enhanced diagnostic precision and decreased empirical prescription. These significant outcomes strongly align with previous findings on diagnostic stewardship. The Bright STAR Collaborative demonstrated similar success, achieving substantial reductions in broad-spectrum antibiotic use (e.g., 13% reduction) alongside lower blood culture rates, without increasing adverse outcomes (7). Our reduction in broad-spectrum agents, including piperacillin/tazobactam (14.12% decrease), corroborates evidence that multidisciplinary initiatives can reduce overall antimicrobial utilization by approximately 10.7% (9).

The substantial reduction in BCER observed in both adult (66.7%) and pediatric (75.0%) emergency departments suggests that implementation of diagnostic stewardship strategies was associated with more selective and appropriate blood culture ordering. Furthermore, the marked reduction in blood culture contamination events (85.7%) highlights the potential benefit of standardized specimen collection procedures and targeted educational interventions. The increase in documentation-based appropriateness from 70 to 97% further suggests improved adherence to institutional guidelines and greater awareness of appropriate blood culture indications among healthcare providers.

A key finding supporting the success of the intervention is the demonstration of safety, with no blood culture-related readmissions reported among the 30-day ED readmissions monitored during the study period. This assurance is consistent with large-scale stewardship studies, including the Bright STAR collaborative study, which confirmed that reductions in BCx utilization did not lead to increases in sepsis, septic shock, mortality, length of stay, or readmissions. Similarly, DS programs among adult non-neutropenic inpatients reported no significant changes in the 30-day mortality or readmission rates. The contextual relevance of these findings lies in their application to the high-throughput ED environment, which is characterized by rapid decision-making and high patient volume, conditions that often lead to defensive ordering and diagnostic variability. The successful implementation of algorithms and standardized protocols at our institute confirms that effective DS is achievable even in challenging, high-stake settings.

## Limitations and future directions

This single-center pre-post design limits the causal inference and generalizability. The short-term follow-up questions long-term sustainability, and the gap between documented and genuine blood culture appropriateness in adults highlights the persistent defensive ordering. Future efforts should integrate diagnostic algorithms into EHRs for real-time decision support. Leveraging artificial intelligence for sepsis prediction can further reduce unnecessary testing. External validation of such tools is crucial. Sustaining these gains requires ongoing staff education and monitoring of safety metrics to ensure that optimized blood culture practices continue to enhance diagnostic precision and antimicrobial stewardship without compromising patient care.

## Conclusion

In this single-center quasi-experimental study, implementation of a multidisciplinary diagnostic stewardship intervention was associated with reductions in blood culture utilization, contamination events, and broad-spectrum antimicrobial consumption in adult and pediatric emergency departments. These findings suggest that structured stewardship strategies may support more appropriate diagnostic testing and antimicrobial use. However, because of the pre-post study design and absence of a control group, causal relationships cannot be established. Future multicenter studies with longer follow-up periods are needed to evaluate sustainability and generalizability.

## Data Availability

The datasets presented in this study can be found in online repositories. The names of the repository/repositories and accession number(s) can be found in the article/supplementary material.
